# Microbiome–Maternal Tract Interactions in Women with Recurrent Implantation Failure

**DOI:** 10.3390/microorganisms13040844

**Published:** 2025-04-07

**Authors:** Manish Kumar, Yang Yan, Luhan Jiang, Ching-Ho Sze, Suranga P. Kodithuwakku, William S. B. Yeung, Kai-Fai Lee

**Affiliations:** 1 Department of Obstetrics and Gynaecology, School of Clinical Medicine, LKS Faculty of Medicine, The University of Hong Kong, Hong Kong SAR 999077, China; manish3@connect.hku.hk (M.K.); jessicaj@hku.hk (L.J.); owens@hku.hk (C.-H.S.); wsbyeung@hku.hk (W.S.B.Y.); 2 Shanghai Key Laboratory of Female Reproductive Endocrine Related Diseases, Obstetrics and Gynaecology Hospital, Fudan University, Shanghai 200032, China; yanyang2022@hotmail.com; 3 Department of Animal Science, Faculty of Agriculture, The University of Peradeniya, Peradeniya 20400, Sri Lanka; surangap@agri.pdn.ac.lk; 4 Institute of Veterinary Medicine and Animal Sciences, Estonian University of Life Science, 51014 Tartu, Estonia; 5 Shenzhen Key Laboratory of Fertility Regulation, The University of Hong Kong, Shenzhen Hospital, Shenzhen 518053, China

**Keywords:** endometrium, hormones, *Lacticaseibacillus rhamnosus*, metabolites, recurrent implantation failure

## Abstract

Microorganisms play an important role in regulating various biological processes in our bodies. In women, abnormal changes in the reproductive tract microbiome are associated with various gynecological diseases and infertility. Recent studies suggest that patients with recurrent implantation failure (RIF) have a reduced genus *Lactobacillus* population, a predominant bacterial species in the vagina and uterus that protects the reproductive tract from pathogenic bacterial growth via the production of various metabolites (e.g., lactic acid, bacteriocin, and H_2_O_2_). Moreover, a higher percentage of pathogenic bacteria genera, including *Atopobium*, *Gardnerella*, *Prevotella*, *Pseudomonas*, and *Streptococcus*, was found in the uterus of RIF patients. This review aimed to examine the role of pathogenic bacteria in RIF, determine the factors altering the endometrial microbiome, and assess the impact of the microbiome on embryo implantation in RIF. Several factors can influence microbial balance, including the impact of extrinsic elements such as semen and antibiotics, which can lead to dysbiosis in the female reproductive tract and affect implantation. Additionally, probiotics such as *Lacticaseibacillus rhamnosus* were reported to have clinical potential in RIF patients. Future studies are needed to develop targeted probiotic therapies to restore microbial balance and enhance fertility outcomes. Research should also focus on understanding the mechanisms by which microorganisms generate metabolites to suppress pathogenic bacteria for embryo implantation. Identifying these interactions may contribute to innovative microbiome-based interventions for reproductive health.

## 1. Introduction

The composition of microorganisms in the vaginal and endometrial microbiome is clinically relevant, as it has been shown to play a role in recurrent implantation failure (RIF) and recurrent pregnancy loss (RPL) [1]. Recurrent implantation failure can be defined as the failure to achieve a clinical pregnancy after at least three in vitro fertilization (IVF) attempts with the transfer of good-quality embryos [2,3,4]. It has a multifactorial etiology involving paternal and maternal factors, embryo quality, and assisted reproductive technology (ART) processes. In addition to the financial burden, RIF can also pose significant psychological burdens on patients undergoing IVF [5]. Clinically, RIF has been associated with uterine structural abnormalities, coagulation abnormalities, and immunological issues [6]. Recently, studies have shown that the composition of the uterine microbiome in IVF patients can also affect pregnancy outcomes [7]. In line with this, a lower pregnancy rate was observed in germ-free mice compared to mice with a normal microbiome [8]. In healthy women, the genital tract is typically colonized by *Lactobacillus* species, including *Lactobacillus crispatus* (*Lb. crispatus*), *Lactobacillus gasseri* (*Lb. gasseri*), *Lactobacillus jensenii* (*Lb. jensenii*), and *Lactobacillus iners* (*Lb. inners*) [9,10]. The naming of genus *Lactobacillus* has been recently updated [11,12]. Patients with a *Lactobacillus*-dominant (LD) (≥90%) vaginal microbiota (VM) were found to have higher implantation and pregnancy rates compared to women with a non-*Lactobacillus*-dominant (NLD) condition [13]. Similarly, RIF patients had a lower abundance of genus *Lactobacillus* compared to the control group (76.4% vs. 91.8%, respectively) [14]. Moreover, the abundance of genus *Gardnerella* in the endometrium of IVF patients was associated with clinical miscarriage [13]. Notably, *Gardnerella vaginalis* (*G. vaginalis*) has been recognized as a causative agent of bacterial vaginosis (BV), endometritis, and pelvic inflammatory disease (PID) [15]. In a study involving 392 RIF patients (216 with a vaginal LD condition and 176 with an NLD condition), there was a higher prevalence of genera including *Bifidobacterium*, *Gardnerella*, *Atopobium*, *Streptococcus*, and *Prevotella* in the RIF group, suggesting the composition of microbiome in the reproductive tract may affect embryo implantation and pregnancy outcomes in IVF treatment [16].

The identification of pathogenic bacteria within the female reproductive tract remains a challenging aspect in optimizing IVF success. Given the complexity of microbial interactions, a comprehensive approach is necessary to improve predictive accuracy. In addition to detecting pathogenic species, external factors such as the semen microbiome and indirect influences such as stress must also be considered. These elements can significantly impact microbial composition, thereby affecting reproductive outcomes. An integrative analysis of these variables could enhance the precision of the assessment of the reproductive microbiome, ultimately contributing to improved diagnostic and therapeutic strategies in ART. Moreover, the association between female disease conditions and IVF success is also complex and is influenced by various reproductive health factors. Studies have identified specific conditions and biomarkers linked to IVF outcomes, which highlights the need to understand their relationships to enhance fertility treatments. Endometriosis, a chronic inflammatory disorder, significantly impacts IVF success. Han et al. (2022) reported that affected women often experience lower clinical pregnancy and egg retrieval rates due to inflammatory cytokines in the follicular fluid that impair embryo quality [17]. These findings underscore the key role of endometriosis in reducing IVF efficacy and its relevance in ART [17]. Therefore, it is crucial to identify pathogenic bacteria and their interactions with internal and external factors in the reproductive tract, which may directly or indirectly influence IVF success in RIF patients.

**Figure 1:**
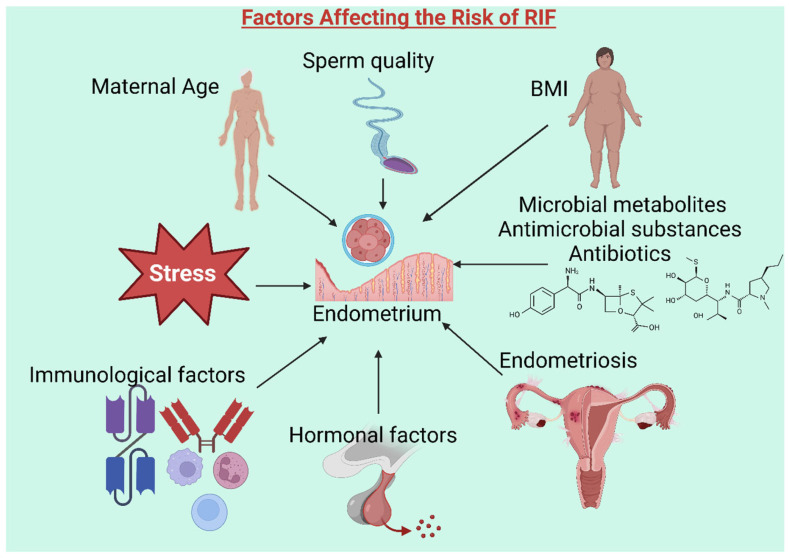
Factors contributing to recurrent implantation failure (RIF). Multiple factors are associated with RIF, including maternal age, body mass index (BMI), microbial metabolites, endometriosis, hormonal and immunological factors, stress, and sperm quality. These elements can influence embryo–endometrium cross-talk and implantation outcomes. (Created with BioRender).

This review aims to summarize the factors affecting the vaginal and endometrial microbiome and gut–uterus axis (Figure 1), which can impact embryo implantation and pregnancy outcomes in RIF patients undergoing ART treatment. Understanding these associations could enhance reproductive care strategies.

## 2. Methodology

This narrative review was conducted in three stages: literature search, abstract and full-text review, and results synthesis. A comprehensive search of relevant studies aligning with the review’s objectives was performed across multiple databases, including PubMed, the HKU library database collection (Biomed Central, PubMed Central, Springer Nature, ASM Journal, and MEDLINE), Scopus, Science Direct, Web of Science, LPSN, Scite, and Google Scholar. The final search was completed in March 2025 and included English-language articles, online reports, and electronic books. The primary keyword “microbiome” was combined with terms such as vaginal and endometrial microorganisms to refine the selection. Abstracts were screened to verify relevance and to remove duplicate entries. Studies meeting the inclusion criteria and analyzing microorganisms in at least two dimensions, such as their presence and potential impact, were selected. Studies focusing on endometrial and vaginal microorganisms were systematically summarized and synthesized to construct this review paper.

## 3. Paternal Factors on the Microbiome

The male microbiome composition can affect the functionality of the female reproductive microbiome. For instance, Luecke et al. investigated the interplay between the seminal microbiome and the vagino-uterine microbiome, given that the seminal and virginal microbiomes share 85% of phylotypes. They found that the microbial communities from both genders can dynamically interact and potentially influence fertilization and implantation processes [18,19]. In human semen, the most common bacterial phyla (*genera*) are Actinobacteria (genus *Corynebacterium*), Bacteroidetes (genus *Prevotella*), Firmicutes (genera such as *Lactobacillus*, *Streptococcus*, and *Staphylococcus*), Planococcaceae (genus *Finegoldia*), and Proteobacteria (genera *Haemophilus* and *Burkholderia*). Genus *Prevotella* was more abundant in semen samples with normal spermiogram parameters [20,21]. In addition, men with abnormal sperm concentration showed a higher abundance of *Pseudomonas stutzeri* (*P. stutzeri*; 2.1% vs. 1.0%, *p* = 0.024) and *Pseudomonas fluorescens* (*P. fluorescens*; 0.9% vs. 0.7%, *p* = 0.010), but a lower abundance of *Pseudomonas putida* (*P. putida*; 0.5% vs. 0.8%, *p* = 0.020) when compared to those with normal sperm concentration [22]. On the other hand, genus *Staphylococcus* was enriched in normospermic samples and genus *Lactobacillus* was enriched in sperm with normal morphology [23]. Notably, *Lactobacillus* was also observed in normospermic samples and was shown to have a positive influence on the vaginal ecosystem [24]. Various pathogenic bacteria, including genera *Gardnerella*, *Prevotella*, and *Ureaplasma*, specifically *G. vaginalis*, and *Prevotella bivia* (*P. bivia*), can induce inflammatory cytokines in the cervicovaginal region [25]. For example, *Gardnerella vaginalis* is a prevalent anaerobic bacterium that causes BV, whereas genus *Ureaplasma* causes genital infections and urinary tract infections (UTIs) in women. In line with this, there was a greater abundance of endometrial genera *Gardnerella*, *Prevotella*, *Atopobium*, *Megasphaera*, *Schlegelella*, *Delftia*, *Burkholderia*, *Sphingobacterium*, *Dietzia*, *Enterococcus*, *Micrococcus*, *Ralstonia*, *Leucobacter*, and *Hydrogenophaga* in patients with RIF (*N* = 145) compared to the control group (*N* = 21) [26].

The diverse array of bacteria within the male reproductive system can lead to various infections and diseases, as highlighted in Table 1 and Table 2. Genus *Gardnerella*, particularly *G. vaginalis*, is commonly linked to BV in women, but can also lead to urethritis and other genital infections in men. Genus *Enterococci* has the potential to induce UTIs, which can escalate to bacteremia and endocarditis in severe cases. Genus *Streptococci* can cause various infections ranging from streptococcal pharyngitis to invasive diseases like pneumonia and sepsis. Genus *Staphylococci*, including *Staphylococcus aureus* (*S. aureus*), is linked to skin infections and abscesses, as well as more severe conditions such as pneumonia and bloodstream infections. Genus *Candida*, particularly *Candida albicans* (*C. albicans*), commonly triggers yeast infections such as balanitis in men. *Escherichia coli* (*E. coli*) is known to cause UTIs, typically affecting the bladder or urethra. Genus *Actinomyces* can lead to infections in the reproductive system such as epididymitis or orchitis.

The varying degrees of severity associated with infections in the male reproductive system highlight the importance of timely medical intervention for accurate diagnosis and effective treatment. For example, oral antioxidant therapies such as lactoferrin and transferrin that inhibit the formation of reactive oxygen species (ROS) and scavenging antioxidants such as vitamins C and E may improve sperm parameters and pregnancy outcomes in patients with oxidative stress and sperm DNA fragmentation [27]. Recently, Su et al. revealed that semen exosomes can facilitate immune evasion of the microbiome in the female reproductive system, suggesting that semen-derived exosomes present during fertilization might play a role in shaping a conducive microbiome setting for embryo implantation [28]. 

## 4. Maternal Factors Affecting the Microbiome

### 4.1. Maternal Age

As women age, there are changes at the molecular, cellular, and histological levels that can affect endometrial receptivity [29]. Advanced maternal age is also strongly associated with higher chromosomal abnormality in the eggs (aneuploidy), which is associated with implantation failure. Women over 40 have a nearly 50% chance of sporadic miscarriage [30]. Notably, there is a decline in microbial diversity with age, with changes in microbiota composition possibly reducing embryo implantation in premenopausal and menopausal women. Shifts in reproductive tract microbiota, alongside hormonal fluctuations, can induce dysbiosis and inflammation, potentially impairing endometrial receptivity [31,32]. In premenopausal women, the VM can be characterized into five different community state types (CST I-V) [33], CST I, CST II, CST III, and CST V, which are primarily dominated by *Lb. crispatus*, *Lb. gasseri*, *Lb. iners*, and *Lb. jenseii*, respectively, and CST IV, which has lower levels of genus *Lactobacillus.* Additionally, CST IV can be further classified into two subtypes: CST IV-A contains anaerobic genera such as *Anaerococcus*, *Peptoniphilus*, *Prevotella*, and *Streptococcus*, whereas CST IV-B contains genera *Atopobium* and *Megasphaera* [33]. Fluctuations in the abundance of these microbiota during menopause can influence the microbiome composition and related vaginal symptoms, including vaginal dryness, vaginal atrophy, and dyspareunia [34]. Furthermore, genus *Atopobium* is reported to be an etiological agent of BV, genus *Prevotella* is also a factor associated with BV and is elevated in the vaginal mucosa, and genus *Streptococcus* is implicated in Streptococcal vaginitis and PID (Table 2). The above findings emphasize the roles of different microbial communities in various pathological conditions, highlighting the intricate relationships between specific bacterial species and the development of gynecological diseases.

### 4.2. Body Mass Index

The body mass index is a measure of an individual’s adiposity or fatness and has a direct and indirect relationship with many reproductive conditions [35]. Women with a BMI > 25 kg/m^2^ have lower embryo implantation rates [36]. A BMI exceeding 30 kg/m^2^ was shown to be significantly associated with an increased risk of implantation failure compared to women with a normal BMI range of 18.5 to 24.9 kg/m^2^ [37]. A meta-analysis comprising 33 studies found that women with a BMI ≥ 25 kg/m^2^ had significantly lower pregnancy and live birth rates compared to those with a BMI < 25 kg/m^2^ following IVF and intracytoplasmic sperm injection (ICSI) treatment [38]. Similarly, a study analyzing data from 22,043 first frozen–thawed embryo transfers concluded that obesity was significantly associated with reduced embryo implantation, clinical pregnancy rates, and live birth rates [39]. In contrast, underweight women exhibited only a slight variation in IVF pregnancy outcomes when compared to those with a normal BMI [39]. Additionally, a study in China published in 2024 found that an elevated BMI was linked to unfavorable pregnancy outcomes after IVF treatment in women with normal ovarian responses [40]. The effects of BMI on pregnancy rate are potentially attributed to an abnormal endocrine, metabolic, and inflammatory environment in the endometrium [41].

Recently, studies have found a significant association between women’s BMI and the reproductive tract microbiome. The microbiome composition in the vagina and uterus differed between women with obesity/overweight and women with a normal BMI [42,43]. The genus *Lactobacillus*-dominant environment in the vagina was compromised in women with increased BMI [42,44]. Moreover, obese women had significantly higher microbial diversity in the uterus and vagina than normal-weight women [42,43]. The uterine microbiota of obese women showed lower levels of genus *Lactobacillus* together with an increased abundance of pathogenic bacteria [43]. The relative abundance of genus *Lactobacillus* in normal-weight women was 2.2 times higher than in overweight and obese women [43]. Healthy-weight women with implantation failure had a higher abundance of pathogenic bacteria in the uterus, including genera *Klebsiella*, *Parasutterella*, *Dialister*, and *Gardnerella*, when compared to healthy-weight women with successful implantation [43]. Moreover, pathogenic bacteria related to adverse pregnancy outcomes were more prevalent in obese and overweight women. The vaginal microbiota of overweight/obese women showed a lower abundance of genus *Lactobacillus* and a trend of higher relative abundance of *Fannyhessea vaginae* (*F. vaginae; formerly Atopobium vaginae*) than that found in normal-weight women, which was linked to increased risk of preterm birth [42].

On the other hand, Blancafort and Llacer suggested that probiotics could regulate BMI and improve reproductive health to enhance fertility outcomes [45]. Additionally, Gille et al. (2023) investigated the effects of probiotics on vaginal health during pregnancy, which were shown to have effects on maternal metabolic health that could potentially influence postpartum BMI [46]. Collectively, these studies suggest that probiotics can modulate microbiota dysbiosis, potentially regulating BMI and improving reproductive health. Further research and clinical trials are needed to elucidate the specific mechanisms through which probiotics may influence BMI and metabolic health.

### 4.3. Smoking

Smoking poses a significant risk for miscarriage in patients undergoing assisted reproduction [47]. Smoking causes ovotoxicity (ovarian toxicity) and decreases estrogen secretion, with women who smoke having lower levels of estradiol during ovarian stimulation [48,49]. Toxic chemicals in cigarette smoke can also disrupt corpus luteum formation and embryo implantation, leading to lower pregnancy rates compared to non-smokers [50].

Notably, smokers were found to have lower levels of vaginal genus *Lactobacillus* compared to non-smokers. Smoking is also a risk factor for BV, which is characterized by reduced genus *Lactobacillus* levels. This has been attributed to the presence of benzo[a]pyrene diol epoxide (BPDE), which has been detected in the vaginal secretions of smokers and was found to promote *Lactobacillus* lysis by bacteriophages [51,52]. Additionally, genus *Lactobacillus* frequently undergoes lysogenization by temperate bacteriophages, and the activation of the lytic cycle may contribute to an unfavorable microbiome shift [53]. Recent studies have shown that tobacco smoke exposure can induce microbial dysbiosis within the vaginal microenvironment, marked by a depletion of lactic acid-producing genus *Lactobacillus* and concomitant enrichment of pathobionts (normal commensal harmless microorganisms) such as genus *Gardnerella* [54]. This dysbiotic state elevates susceptibility to BV and synergistically enhances host vulnerability to viral pathogens, including human papillomavirus (HPV), through compromised mucosal immune homeostasis [55]. Furthermore, smoking-related perturbations in vaginal metabolomics disrupt local inflammatory signaling and epithelial barrier integrity, potentially exacerbating gynecologic morbidities [54].

The impact of smoking on the vaginal microbiome is further shaped by dietary factors, prompting research on how a healthy diet may mitigate the adverse effects [56]. Rosen et al. (2021) emphasized the role of lifestyle choices, including diet, in modulating the relationship between smoking and the microbiome, highlighting the need for further investigations into these modifiable factors and their implications for reproductive health [56].

Future research should prioritize longitudinal studies to clarify the mechanisms by which smoking alters the vaginal microbiome composition and its association with reproductive health outcomes. These studies should integrate multifactorial influences such as hormonal variations and dietary factors to provide a comprehensive understanding of these interactions.

### 4.4. Stress

Stress has been shown to significantly affect the microbiome in the female reproductive tract, potentially influencing reproductive health and outcomes. Cortisol is produced in response to psychological, immunological, and other stressors, which together are not conducive to pregnancy [57]. Women trying to conceive were found to have higher levels of anxiety compared to those who were pregnant, likely due to stressors associated with the fertility treatments, societal pressures, and career challenges, which can exacerbate feelings of anxiety [20]. It has been shown that stress can lead to an imbalanced VM. In mice, stress caused disruptions in the vaginal mucosal- and immune response-related proteins. In humans, early prenatal stress was found to reduce the relative abundance of the genus *Lactobacillus* in the vagina [58]. In addition to affecting the level of genus *Lactobacillus*, stress-related disruptions can also reduce the bactericidal potency of neutrophils, leading to the growth of anaerobic and facultative bacteria [59,60], which can affect embryo implantation and pregnancy outcomes. Moreover, chronic stress has been associated with increased susceptibility to BV.

Stress can also activate the hypothalamic–pituitary–adrenal (HPA) axis, leading to glucocorticoid release, particularly cortisol, which regulates immune function and microbiome stability [61]. Jašarević et al. reported that stress decreased a key antimicrobial protein, lactoferrin, leading to impaired mucosal immunity and heightening infection risk. These findings suggest that stress-induced microbial dysbiosis compromises vaginal health, potentially predisposing individuals to infections through immune modulation and epithelial barrier disruption [62].

### 4.5. Disease

#### 4.5.1. Chronic Endometritis

Chronic endometritis (CE) is the long-term inflammation of the endometrial lining, characterized by the presence of edema, high stromal cell density, dissociated maturation of both stromal and epithelial tissues, and infiltration of plasma cells within the stroma [63]. Importantly, CE can lead to infertility and RPL [64,65], with a reported prevalence of approximately 14% in RIF patients and 27% in RPL patients [64]. Patients with RIF can have minimal or no clinical signs or symptoms of CE, whereas those with CE have an implantation rate of only around 11.5% [66,67]. Several bacteria have been implicated in CE, including Gram-positive bacteria (genera *Streptococcus* and *Staphylococcus*), Gram-negative bacteria (genera *E. coli*, *Klebsiella pneumoniae* (*K. pneumoniae*), and *Neisseria gonorrhoeae* (*N. gonorrhoeae*)), intracellular bacteria (genera *Mycoplasma*, *Ureaplasma*, and *Chlamydiae*), and anaerobic bacteria (genera *Bifidobacteria* and *Prevotella*) [68]. Patients with CE can have diverse microbiome compositions (Table 1), each associated with specific diseases (Table 2), including *Chlamydia trachomatis* (*C. trachomatis*) (causing chlamydia), *E. coli* (implicated in UTIs, PID, vaginal infections, and tubo-ovarian abscess), genus *Mycoplasma* (linked to BV and cervicitis), *N. gonorrhea* (causing gonorrhea), genus *Prevotella* (linked to increased vaginal mucosal characteristics of BV), and genus *Streptococcus* (associated with Streptococcal vaginitis and PID). Removing these bacteria increased pregnancy rates from 33% to 65.2% compared to women with persistent infections. Moreover, patients with CE successfully treated with antibiotics significantly increased their live birth rate from 13.3% to 60.8% [4].

#### 4.5.2. Endometriosis

Endometriosis (EM) is a condition characterized by the presence of endometrial glands and stroma outside the normal lining of the uterus [69]. It affects 9% to 50% of women with infertility problems [69]. Patients with EM have reduced numbers of oocytes and embryos, leading to lowered implantation and pregnancy rates and increased risk of abortion [70]. Notably, patients receiving antibiotic treatments for EM have improved IVF outcomes [71]. A recent study proposed a link between EM development and bacterial contamination in the female reproductive tract [72]. It was hypothesized that inflammatory bacterial lipopolysaccharide (LPS) may trigger the growth of endometriotic tissue [73]. Endometriotic patients were found to have significantly increased bacteria families, including Streptococcaceae, Moraxellaceae, Staphylococcaceae, and Enterobacteriaceae, but genus *Lactobacillus* was significantly decreased [74]. Uterine flushing commonly shows genera *Lactobacillus*, *Barnesiella*, *Flavobacterium*, and *Pseudomonas* [75], but patients with EM showed highly abundant genera *Pseudomonas*, *Acinetobacter*, *Vagococcus*, and *Sphingobium*, suggesting infertile females with EM will also have a distinct uterine microbiota composition [76]. Likewise, compared to normal endometrial tissue (eutopic endometrium), endometriotic lesions exhibited greater microbial diversity, including genera *Lactobacillus*, *Enterococcus*, *Gardnerella*, *Pseudomonas*, *Alishewanella*, *Ureaplasma*, and *Aerococcus* [77].

Probiotics have demonstrated the potential to improve EM-associated pain symptoms. Probiotics were able to reduce endometriotic lesions in animal models, suggesting they have therapeutic value for managing EM [78]. Probiotic supplementation was also associated with positive health effects in individuals with EM, indicating they could serve as an alternative or complementary approach to conventional treatments [79].

**Table 1:** Factors affecting microbiome composition in the reproductive tract of women.

Factors/Sample	Microorganisms	Population/Techniques/Database	Reference
Male Factor			
Urethral and penile skin	Genera *Actinomyces*, *Anaerococcus*, *Atopobium*, *Aerococcus*, *Barnesiella*, BVAB1, BVAB2, *Dialister*, *Eggerthella*, *Gardnerella*, *Gemella*, *Lb. iners*, *Leptotrichia*, *Mycoplasma hominis (M. hominis*), *Parvimonas*, *Peptoniphilus*, *Peptostreptococcus*, *Porphyromonas*, *P. bivia*, *Prevotella disiens* (*P. disiens*), and *Sneathia.*	Male partners of women with and without BV, *N* = 96 couples (96 vaginal, 94 urethral, and 93 penile skin), pyrosequencing.	[80]
Coronal sulcus	Genera *Anaerococcus*, *Corynebacteria*, *Delftia*, *Finegoldia*, *Peptoniphilus*, *Porphyromonas*, *Prevotella*, *Propionibacterium*, and *Staphylococcus.*	Adolescent men, *N* = 18 (12 circumcised, 5 uncircumcised, 1 excluded). Pyrosequencing V1–V3, V3–V5, V6–V9.	[81]
Seminal plasma	Genera *Candida*, *Enterobacteria*, *Enterococci*, *Streptococci*, and *Staphylococci.*	Men of infertile couples, *N* = 71 (all 71 infertile asymptomatic), culture.	[82]
Semen	Genera *Anaerococcus hydrogenalis* (*A. hydrogenalis*), *Acinetobacter johnsonii* (*A. johnsonii*), *A. vaginae*, *Bacteroides ureolyticus* (*B. ureolyticus*, now *Campylobacter ureolyticus*), *Campylobacter rectus* (*C. rectus*), *Corynebacterium seminale (C. seminale*, now *Corynebacterium glucuronolyticum*), *Enterobacter cowanii (E. cowanii*, now *Kosakonia cowanii*), *G. vaginalis*, *Janthinobacterium lividum* (*J. lividum*), *Lb. crispatus*, *Lb. iners*, *Peptostreptococcus anaerobius* (*P. anaerobius*), *Peptostreptococcus asaccharolyticus* (*P. asaccharolyticus*), *Pseudomonas veronii* (*P. veronii*), *Streptococcus infantis* (*S. infantis*), and *Varibaculum cambriense* (*V. cambriense*).	Men with and without prostatitis, *N* = 67 (21 with prostatitis, 46 without prostatitis), sequencing at V6.	[83]
**Female Factor**			
Vaginal	*A. vaginae*, *G. vaginalis*, genus *Lactobacillus*, and *M. hominis.*	IVF patients, *N* = 307 (with BV = 29, without BV = 278), qPCR.	[84]
Cervicovaginal	*C. trachomatis* and *N. gonorrhoeae.*	*N* = 230, pregnant (*N* = 14) and non-pregnant (*N* = 194), PCR.	[85]
Cervix	*C. trachomatis*, genus *Gardnerella*, genus *Lactobacillus*, *N. gonorrhoeae*, genus *Prevotella*, and genus *Sneathia.*	Women with or without infectious infertility, female sex workers and healthy controls, *N* = 190 (26 non-infectious infertility, 21 infectious infertility, 89 fertile and healthy, 54 female sexual workers). Sequencing at V3–V4.	[86]
Endometrial	The endometrial genus *Lactobacillus* levels did not significantly differ between RIF and controls (51.2% ± 37.5% and 51.6% ± 38.3%, respectively). Higher endometrial microbiota in the RIF group than the control group: genera *Atopobium*, *Bacillus*, *Bifidobacterium*, *Corynebacterium*, *Enhydrobacter*, *Exiguobacterium*, *Gardnerella*, *Megasphaera*, *Ochrobactrum*, *Prevotella*, *Pseudoalteromonas*, *Shewanella*, *Streptococcus*, and *Vibrio.*	*N* = 145 (RIF) and *N* = 21 (control).	[26]
Fallopian tubal flushing	Genus *Mycoplasma.*	Patients with tubo-peritoneal infertility and normal fertile patients, *N* = 60. (30 normal, 30 infertile), PCR.	[87]
Follicular fluid	Genera *Actinomyces*, *C. parapsilosis*, *C. aurimucosum*, *Fusobacterium*, *Lb. iners*, *P. asaccharolyticus*, *Peptostreptococcus*, *Prevotella*, *Propionibacterium*, and *Staphylococcus.*	ART patients, *N* = 71 (18 fertile, 16 with endometriosis, 14 with PCOS, 9 genital tract infection, 14 idiopathic infertility), PCR.	[88]
Peritoneal fluid	*Mycoplasma genitalium* (*M. Genitalium*) and *M. hominis.*	Women with and without endometriosis, *N* = 104 (73 with endometriosis, 31 without endometriosis), PCR.	[89]
**Female Age Factor**			
Mid-vaginal	Genera *Aerococcus*, *Anaerococcus*, *Atopobium*, *Dialister*, *Diaphorobacter*, *Finegoldia*, *Lb. crispatus*, *Lb. gasseri*, *Lb. jensenni*, *Lb. iners*, *Megasphaera*, *Parvimonas*, *Peptinophilus*, Proteobacteria, *Prevotella*, *Sneathia*, *Streptococcus*, and *Veillonella.*	Premenopausal women, *N* = 30 (all premenopausal women), sequencing at V1–V2.	[90]
Vaginal	Genera *Atopobium*, *Dialister*, *Gardnerella*, *Lactobacillus*, *Megasphaera*, and *Prevotella.*	Premenopausal women’s vaginal microbiome, *N* = 396 (all vaginal collections), sequencing.	[33]
**Female Disease Factor**			
Chronic Endometritis (CE)	Genera *Bifidobacteria*, *Chlamydia*, *E. coli*, *K. pneumoniae*, *Mycoplasma* *N. gonorrhoeae*, *Prevotella*, *Streptococcus*, *Staphylococcus*, and *Ureaplasma.*	113 patients with CE, sequencing of 16S rRNA V2–4–8, V3–6, V7–9 regions.	[68]
Endometriosis (EM)	EM patients with a decrease in genus *Lactobacillus* and an increase in genera *Pseudomonas*, *Acinetobacter*, *Vagococcus*, and *Sphingobium.* Non-EM: genus *Lactobacillus* dominant.	EM (*N* = 36) and non-EM (*N* = 14) women, sequencing at V4–V5 region.	[91]
**Estrobolome**			
Hormone factor	Genera *Bacteroides*, *Bifidobacterium*, *Clostridium*, *Escherichia*, and *Lactobacillus.*	Human Microbiome Project (HMP) gut-associated microbial genomes (*N* = 517) were indexed for the presence of β-glucuronidase (EC 3.2.1.31). Estrogen is metabolized by the β-glucuronidase between conjugate forms to the deconjugate form.	[92]
**Antibiotic Factor**			
Metronidazole	Used for treatment of bacterial vaginosis. Effective against protozoa, *Bacteroides fragilis* (*B. fragilis*), *Clostridium difficile* (*C. difficile*, now *Clostridioides difficile*), and genus *Fusobacterium*, less effective against *Lactobacillus* strains.	*N* = 392 (all diagnosed with RIF).	[16]
**Stress Factor**			
Chronic stress	Stressed females had increased Proteobacteria at gestation day 7.5, mainly from the *Helicobacter* genus. Stress reduced genus *Lactobacillus* in females at postnatal day 2.	Control (*N* = 5), treatment group (*N* = 8).	[58]

**Table 2:** Microorganisms and the associated diseases/infection.

Microorganisms	Pathological Condition	Reference
*Acinetobacter*, *E. coli*	Postpartum endometritis	[93]
*Aerococcus urinae* (*A. urinae*)	Urinary tract infections (UTIs)	[94]
*Anaerococcus*	Various infections (vaginal discharge, ovarian abscesses, skin infections, chronic wounds)	[95,96]
*Atopobium*, *Bacteroides*	Bacterial vaginosis (BV)	[97]
*B. fragilis*	Pelvic inflammatory disease (PID)	[98]
*C. trachomatis*	*Chlamydia trachomatis*, the bacterium, is associated with the sexually transmitted disease, Chlamydia.	[99]
*Clostridium perfringens* (*C. perfringens*)	Gas gangrene	[100]
*Desulfovibrio microaerophilic* (*D. microaerophilic*)	Gynecological infections (Pyometra)	[101]
*E. coli*	UTIs, PID, vaginal infections	[102,103,104]
*E. coli*, *B. fragilis*, *Peptostreptococcus*	Tubo-ovarian abscess	[105]
*G. vaginalis*	BV	[106]
*Haemophilus ducreyi* (*H. ducreyi*)	Chancroid	[107]
Herpes simplex virus	Genital herpes (STD)	[108]
Human immunodeficiency virus (HIV)	Acquired immunodeficiency syndrome (STD)	[109]
*M. genitalium*, *N. gonorrhoeae*	PID	[110,111]
*M. hominis*	BV, cervicitis and endometritis	[112,113]
*N. gonorrhoeae*	Gonorrhea (STD)	[114]
Papillomavirus	Human papillomavirus (STD), genital warts, abnormal cervical cell changes, and an increased risk of cervical cancer	[115]
*Prevotella*	Increased abundance in vaginal mucosa is associated with BV	[116]
*Propionibacterium*	Chronic endometritis, BV(sometimes detected)	[68]
*Pseudomonas aeruginosa* (*P. aeruginosa*)	PID, UTIs	[117,118]
*Sneathia amnii* (*S. amnii*)	Bartholin’s gland cyst and also linked with BV, preterm labor, chorioamnionitis, stillbirth and peripartum bacteremia	[119,120,121]
*Streptococcus*	Streptococcal vaginitis, PID	[122,123]
*S. aureus*	PID, vaginitis, endometritis, Bartholin’s gland abscess	[124,125,126,127]
*Trichomonas vaginalis* (*T. vaginalis*)	Trichomoniasis	[128]

### 4.6. Gut Epithelial Integrity

The gut–uterus axis represents a significant area of research that explores the bidirectional communication between the gut microbiome and the female reproductive system. Emerging research indicates that the gut microbiota can influence systemic inflammation and immune responses, which are critical factors in the implantation process. Patel et al. (2021) showed that women experiencing RIF and unexplained infertility had distinct gut and vaginal microbiota profiles, suggesting that the composition of these microbiota could play a role in reproductive outcomes [129]. Furthermore, alterations in gut microbiota were found to affect the regulation of immune system components essential for successful implantation. Blazheva et al. (2024) showed that women with disrupted endometrial microbiota displayed lower levels of immune cells, including uterine natural killer (uNK) cells, which are crucial for maintaining a receptive endometrial environment. The depletion of key immune regulators may contribute to implantation failure [31]. Additionally, the gut microbiota is known to influence the systemic immune response, potentially impacting the uterine environment and receptivity [31]. Nevertheless, other mechanisms may also be involved, such as dysbiosis of the gut microbiota, which can lead to increased levels of inflammatory cytokines, adversely affecting reproductive health. High levels of TNF-α and other pro-inflammatory cytokines can affect embryo implantation by creating a hostile environment for embryo reception [129]. In addition to the gut–uterus axis, the gut–brain axis can also contribute to changes in gut microbial profiles, leading to altered hormonal and immune responses that further complicate implantation [129].

Activated T cells and NK cells can trigger the release of pro-inflammatory cytokines, leading to the formation of blood clots at the fetal–maternal interface in RIF patients [130]. In line with this, RIF patients were shown to have elevated levels of IL-1 β, IL-6, and TNF-α [131]. In normal pregnancy, Th-1 cytokines such as IL-2, IL-3, INF-γ, and TNF-α and β are downregulated, whereas Th-2 cytokines such as IL-4, IL-5, IL-6, IL-9, IL-10, and IL-13 are upregulated [132,133]. Recent studies suggest that *Megasphaera elsdenii* (*M. elsdenii*) and *Hoylesella timonensis* (*H. timonensis;* formerly *Prevotella timonensis*), which are commonly found in the human gastrointestinal tract and sometimes in RIF patients, can induce the release of pro-inflammatory cytokines, including IL-1β, IL-6, and TNF-α. Dendritic cells exposed to vaginal bacteria *M. elsdenii* and *P. timonensis* produced IL-1β, IL-6, IL-8, IL-12p40, and TNF-α cytokines [14]. Moreover, TNF-α production can also be stimulated by LPS and peptidoglycan from bacteria including *E. coli* and *Limosilactobacillus mucosae* (*Lm. mucosae*) [134]. In inflammation-induced preterm labor, TNF-α was found to induce uterine smooth muscle cell collagen contractility [135]. In mice, bacterial LPS-induced TNF-α production resulted in implantation failure [136].

### 4.7. Hormones

Estrogen and progesterone play crucial roles in endometrial growth and differentiation in preparation for embryo implantation [137]. For example, estrogen regulates endometrial cell proliferation, glycogen storage, and mucus secretion. In humans, the composition of endometrial microbiota is also regulated by hormones [23]. Estrogen regulates vaginal pH by increasing lactobacilli abundance and lactic acid production [138]. The estrobolome is a group of bacteria that can metabolize estrogen [139]. In the gut, estrogen is metabolized by β-glucuronidase (deconjugates estrogens into their active forms), which is found in genera *Bacteroides*, *Bifidobacterium*, *Clostridium*, *Escherichia*, and *Lactobacillus* [92,139]. However, a reduction in circulating estrogen can lead to hypoestrogenic pathologies such as obesity, metabolic syndrome, cardiovascular disease, and cognitive decline. Obesity and metabolic syndrome are also associated with polycystic ovary syndrome, endometrial hyperplasia, and infertility [140,141]. Interestingly, the administration of estrogen and progesterone in RIF patients can promote a synchronized uterine environment that is conducive to embryo implantation [142,143].

Estrogen is also essential for vaginal microbiome stability, as it regulates glycogen storage, which serves as a key nutrient for *Lactobacillus* species. Wu et al. (2021) reported that estrogen fluctuations, particularly during reproductive years, significantly influence microbiome composition. A decline in estrogen during menopause was shown to reduce genus *Lactobacillus* diversity and increase pathogenic species, contributing to vaginal atrophy and dryness [144]. Furthermore, Micks et al. (2023) showed that declining estrogen levels during menopause leads to changes in vaginal pH and microbiome composition, which suggests estrogen therapy could modulate the vaginal microbiome and alleviate menopause symptoms such as genitourinary syndrome, although microbial regulation may not always correlate with clinical symptom relief [145]. Zhao et al. (2020) showed that elevated estrogen levels during procedures like in vitro fertilization could impact VM composition. They found that estrogen enhanced *Lactobacillus* growth in a glycogen-rich environment, contributing to microbiome stability and reproductive success [146].

## 5. Bacterial Metabolites and the Microbiome

### 5.1. Short-Chain Fatty Acids

Metabolic molecules have direct and indirect effects on physiological and pathophysiological processes in the body. Short-chain fatty acids (SCFAs) are carboxylic acids with two to six carbon atoms and an aliphatic tail. They are mainly generated in the liver through metabolic processes, but can also be synthesized by bacteria, including members of the Bifidobacteriaceae family (e.g., genus *Bifidobacterium*), in the colon and uterus. Bacterial SCFAs have been detected on mucosal surfaces including the oral cavity (1–16 mM) and the intestinal tract (70–140 mM) [147,148]. Gut microbes can mainly form acetate, propionate, and butyrate SCFAs, which are believed to play a role in regulating the immune system of the gastrointestinal tract [149], as well as modulate oxidative burst, degranulation, and phagocytic functions [150,151,152]. Furthermore, epithelial cells utilize SCFAs as an energy source, regulating cell proliferation, differentiation, and apoptosis [153,154,155]. The administration of maternal short- and medium-chain fatty acids 0.1% sodium butyrate (SB), 0.1% sodium hexanoate (SH), or 0.1% sodium caprylate (SC)] was observed to enhance pregnancy outcomes and promote successful embryo implantation. Some studies in rats demonstrated that maternal supplementation with SB, SH, or SC significantly elevated the number of embryo implantation sites by 2.53, 2.64, and 2.59 pups, respectively. The maternal supply of short- and medium-chain fatty acids in rats was shown to increase the number of embryo implantation sites by ensuring a balanced spacing between embryos, ultimately leading to improved pregnancy outcomes [156]. Moreover, SCFAs can decrease bacterial phagocytosis by neutrophils through an FFAR2-independent mechanism [157]. For example, the opportunistic pathogenic bacterium *Aggregatibacter actinomycetemcomitans* (*A. actinomycetemcomitans*) can translocate from the oral cavity to the gut, potentially disrupting microbial balance and promoting inflammation, while inoculation leads to the production of pro-inflammatory cytokines (e.g., TNF-α, IL-1β, IL-6, and IL-12), chemokines (e.g., Cxcl1 and Cxcl2), and anti-inflammatory cytokine IL-10. Similarly, the exposure of preimplantation stage mouse embryos to high levels of maternal TNF-α may lead to implantation failure and poor pregnancy outcomes [137,157]. This indicates that SCFAs may impair host immunity by inhibiting neutrophil effector functions.

Furthermore, SCFAs, such as acetate, propionate, and butyrate, produced by the gut microbiota through the fermentation of dietary fibers, play crucial roles in host metabolic health [158,159]. Research suggests that SCFAs can influence reproductive outcomes by modulating reproductive microbiota and systemic metabolism. Zeng et al. reported that short- and medium-chain fatty acids enhanced ovarian steroidogenesis and endometrial receptivity in sows, suggesting their potential role in optimizing pregnancy conditions in humans [160]. Moreover, SCFAs were shown to have anti-inflammatory properties and promote immune tolerance, both essential for pregnancy [161]. Hu et al. demonstrated that maternal SCFAs alleviated oxidative stress and regulated inflammation through gut microbiota interactions, underscoring their importance in pregnancy outcomes [161]. Hence, a microbiome enriched with SCFAs can support metabolic adaptations and reduce the risk of infection, contributing to fetal growth. Additionally, SCFAs can regulate maternal metabolism to meet the energy demands of both mother and fetus [162]. Liu et al. highlighted their role in immune cell modulation, which is critical for pregnancy maintenance [163]. Chen et al. found that reduced SCFA production due to dysbiosis was associated with complications such as gestational diabetes and preeclampsia, emphasizing their role in preventing pregnancy-related disorders [164].

### 5.2. Endogenous Antimicrobial Substances

Estrogen plays a crucial role in modulating immune responses in the endometrial epithelium during the proliferative phase of the menstrual cycle in humans. Estrogen has anti-inflammatory effects on the uterine environment by reducing pro-inflammatory cytokines such as TNF-α, IL-1β, IL-6, and IL-8 [165,166]. Estrogen also plays a role in local immune defense by stimulating the production of antimicrobial peptides, including secretory leukocyte peptidase inhibitor (SLPI) and β-defensin 1–2 (HBD 1–2) [165,166]. Estrogen significantly induced SLPI expression in endometrial epithelial cells in rats [167].

Notably, SLPI and TNF-α have opposing roles in immune regulation, with SLPI suppressing and TNF-α promoting inflammation. This inverse relationship is further supported by the finding that IFN-γ inhibits SLPI while enhancing TNF-α expression in murine macrophages exposed to apoptotic cells [168]. Elevated TNF-α levels are strongly associated with pregnancy complications, including recurrent miscarriage, preeclampsia, premature membrane rupture, and intrauterine growth restriction, primarily due to its roles in trophoblast apoptosis and inflammation [169]. In Lewis lung carcinoma, TNF-α was found to induce SLPI expression, suggesting a feedback mechanism where SLPI modulates TNF-α’s inflammatory effects [170]. Endometrial SLPI levels increase during the menstrual cycle, indicating it plays a role in regulating local immune responses [171]. Moreover, SLPI may have pro-inflammatory properties by suppressing the differentiation of Treg cells, which are crucial for maintaining immune homeostasis and tolerance [172]. Studies showed that Treg depletion can lead to implantation failure and fetal resorption. Correspondingly, immunosuppressive therapy with anti-TNF antibodies or tacrolimus was found to improve pregnancy outcomes in women with RIF and RPL, in humans [173]. In mice, Tregs were also implicated in successful pregnancy [174].

The vaginal microbiome is dominated by genus *Lactobacillus*, which plays a crucial role in protecting against infections and supporting reproductive health. These bacteria help maintain an acidic pH, creating a barrier against pathogens and reducing the risk of dysbiosis, which has been linked to bacterial vaginosis and adverse pregnancy outcomes such as preterm birth [175]. Beneficial microbes such as *Lactobacillus* can also enhance antimicrobial effectiveness by competing with pathogenic bacteria, thereby minimizing infections during pregnancy [176]. However, antimicrobial treatments can alter microbiome composition and function, and antibiotic exposure during pregnancy may increase the risk of complications such as preterm birth and gestational diabetes [177]. Chen et al. reported that disruptions to the gut microbiome during pregnancy could elevate the risk of conditions like preeclampsia, highlighting the importance of the microbiome on the maternal immune response to infections and treatments [178]. Moreover, the ecological dynamics of the microbiome during pregnancy can be influenced by diet, which plays an essential role in modulating both microbial composition and the activity of antimicrobial substances. A fiber-rich diet (balanced diet) promotes the growth of beneficial microbes that produce SCFAs, which have anti-inflammatory properties and may contribute to positive pregnancy outcomes [179]. In short, the interplay between antimicrobial agents, microbiome health, and pregnancy outcomes is complex. Microbial dysbiosis can alter infection susceptibility and cause pregnancy complications, emphasizing the need for therapeutic strategies that safeguard microbiome integrity, while ensuring maternal and fetal health.

### 5.3. Antibiotics

Antibiotics are pharmacological agents commonly used to suppress the growth of microorganisms. Antibiotics are commonly used during pregnancy to manage infections that, if untreated, may lead to complications such as preterm birth and low birth weight [180]. A recent study suggested that a combination of vaginal probiotic suppositories and antibiotics may increase the proportion of the beneficial bacteria genus *Lactobacillus.* Notably, among the genus *Lactobacillus* in pregnant women, *Lb. crispatus* exhibits the highest prevalence (100%), followed by *Lb. iners* (77%), *Lb. jensenii* (74%), and *Lb. helveticus* (60%) [181,182]. Treatment with antibiotics was shown to improve pregnancy outcomes of women with CE by removing infection-causing bacteria [68,183].

However, animal and human studies have demonstrated that early-life antibiotic exposure increases BMI, induces overweight and central adiposity, and negatively affects embryo implantation [184,185]. Moreover, antibiotics can disrupt the vaginal and gut microbiota, potentially affecting pregnancy outcomes. Dunlop et al. reported that antibiotic exposure alters the VM, influencing microbial stability throughout pregnancy [186]. Antibiotic-induced disruptions to the VM may reduce protective microbial populations, increasing susceptibility to infections and complications [187]. Certain VM profiles are associated with preterm birth risk, and disruptions to the VM can lead to dysbiosis, which has been linked to adverse outcomes, including preterm birth [187]. Prenatal antibiotic exposure has also been associated with long-term health risks in children, such as asthma and obesity, likely due to microbiome dysbiosis [188]. DiGiulio et al. highlighted that antibiotics administered during critical microbiome development stages can have lasting effects on microbial composition and function, impacting maternal and infant health [189]. Gut microbiota alterations can also influence metabolite production, affecting immune responses and inflammation, and further complicating pregnancy outcomes [190]. The gut microbiome plays a key role in maternal and fetal health by producing metabolites like SCFAs that have anti-inflammatory properties and support fetal development. Antibiotic-induced dysbiosis may disrupt these protective factors, increasing pregnancy risks [191]. In short, antibiotics are essential for infection management during pregnancy, but their impact on microbiome health necessitates cautious prescribing practices. Understanding microbiome–antibiotic interactions is crucial for minimizing risks and optimizing maternal and fetal health.

## 6. Microbiome Modulation in Patients with Recurrent Implantation Failure

A recent study found that about 44.9% of RIF patients exhibited NLD microbiota [16]. However, research on manipulating the composition of endometrial microbiota to favor the growth of genus *Lactobacillus* remains limited. Furthermore, interactions of the vaginal and uterine microbiome may also play a role in regulating fertility in humans [181]. Further investigations are needed to understand the role of microbiome–maternal tract interactions. In addition to boosting the abundance of genus *Lactobacillus*, eliminating pathogens from the microbiota may also enhance implantation. For example, given that BV is frequently detected in NLD cases, the administration of metronidazole (a primary drug for BV that is effective against genus *Lactobacillus*) becomes imperative in infertile patients [16].

## 7. Future Prospectives

The role of probiotics such as genera *Lactobacillus* and *Bifidobacterium* on reproductive health depends on their specific biological functions [192]. In males, the use of probiotics and synbiotics (combinations of probiotics and prebiotics) is associated with enhanced semen quality [193,194]. In females, probiotics have been observed to have a protective role against vaginal infections [195,196]. Oral administration of genus *Lactobacillus* in women with EM was found to be beneficial in reducing EM-related pain [2]. Recent research has shown that *Lacticaseibacillus rhamnosus* (*Ls. rhamnosus*) can lower pH levels, generate SCFAs, provide protection against pathogen colonization, and facilitate lactate production [197]. *Bifidobacterium* (SCFA producer) probiotic supplementation in mice enhanced placental function and fetal development, indicating contribution to favorable pregnancy outcomes [198]. Another study showed that *Limosilactobacillus reuteri* (*Lm. reuteri*) and *Ls. rhamnosus* can enhance the endometrial epithelial barrier in response to human immunodeficiency virus-1 (HIV-1) infection [199].

Prebiotics (compounds in food that foster the growth or activity of beneficial microorganisms) such as lactoferrin can be orally administered during and after the use of antibiotics in women undergoing infertility treatment [200]. In patients with a low abundance of genus *Lactobacillus*, 3 months of lactoferrin treatment following antibiotic therapy resulted in a high abundance of genus *Lactobacillus* in the endometrium in two-thirds of subjects [200]. Furthermore, the use of lactoferrin has shown promise in combating BV, resulting in more successful pregnancies and full-term births in women with a prior history of preterm delivery [201].

Future studies are needed to develop probiotic therapies for reproductive health, with a particular focus on restoring microbial balance to enhance fertility outcomes.

## 8. Conclusions

Vaginal microbiota play an important protective role within the female reproductive tract by regulating various biological processes. The presence of genus *Lactobacillus* in the vagina and uterus favors pregnancy outcomes. However, patients with RIF showed genus *Prevotella* (associated with premenopausal women and obese women) and genus *Staphylococcus* (associated with higher BMI and lower embryo implantation rates), whereas patients with CE showed pathogenic genera *Staphylococcus*, *Prevotella*, and *Streptococcus*. Furthermore, the production of pro-inflammatory cytokines such as IL-1β and IL-6 by *P. timonensis* and *M. elsdenii* was associated with RIF. Conversely, SCFAs generated by *Ls. rhamnosus* provide protection against pathogen colonization and facilitate lactate production conducive to embryo implantation. The findings show that dysbiosis of the reproductive microbiome is associated with various gynecological diseases and infertility, while probiotics could restore balance and improve reproductive health.

## Data Availability

No new data were created or analyzed in this study.

## Abbreviations

ART	assisted reproductive technology
BMI	body mass index
BV	bacterial vaginosis
CE	chronic endometritis
CSTs	community state types
EM	endometriosis
HMP	human microbiome project
IVF	in vitro fertilization
LD	*Lactobacillus* dominant
LPS	lipopolysaccharide
NLD	non-*Lactobacillus* dominant
PID	pelvic inflammatory disease
RIF	recurrent implantation failure
RPL	recurrent pregnancy loss
SCFAs	short-chain fatty acids
STD	sexually transmitted disease
UTIs	urinary tract infections
VM	vaginal microbiota
